# Parity and nutrient total-tract digestibility in dairy cows during transition period

**DOI:** 10.1016/j.vas.2023.100313

**Published:** 2023-09-06

**Authors:** Julio de M. Vettori, Damiano Cavallini, Melania Giammarco, Lydia Lanzoni, Oreste Vignone, Hassan Jalal, Ludovica Mammi, Paolo Pezzi, Andrea Formigoni, Isa Fusaro

**Affiliations:** a Dipartimento di Scienze Mediche Veterinarie, University of Teramo, Teramo, Italy; b Dipartimento di Scienze Mediche Veterinarie, Alma Mater Studiorum University of Bologna, Bologna, Italy

**Keywords:** Parity, Transition period, Dairy cows, Total-tract digestibility

## Abstract

Cows experience many physiological, nutritional and social changes during transition period. This study aims to evaluate the differences in nutrients total-tract digestibility (nTTD) between primiparous (PP) and multiparous (MP) cows through the transition period. From -23, -5, 0, 7, 14, and 30 days from calving a sample of feed and feces samples were collected from 25 Holstein cows (11 PP and 14 MP) as well as daily rumination time through accelerometers.

The results show that average nTTD were different (*p* ≤ 0.02) for amylase-treated neutral detergent fiber organic matter (aNDFom) and potentially digestible neutral detergent fiber (pdNDF_240_; 52.5 vs. 54.0 and 78.8 vs. 81.3, respectively in PP vs. MP), while no differences were found regarding pdNDF_24_ and starch (88.5 vs. 88.6 and 95.1 vs. 96.1, respectively in PP vs. MP). Total-tract starch digestibility (TTstarchD) was different among timepoints (*p <* 0.01), going from an average of 91.40 up to 97.39% of starch, on times -23 and 14, respectively. Differences in total-tract digestibility of aNDFom among timepoints (*p <* 0.01) was expected because of differences in diet composition among lactating and non-lactating cows. No differences in daily rumination time (*p* = 0.92), TTstarchD and total-tract potentially digestible NDF digestibility at 24 h (TTpdNDF_24_D) were recorded. Our findings show that fiber digestibility during the transition period is higher in MP probably for a different ruminal retention time. These differences should be considered when formulating rations for groups with different parity number.

## Highlights

• Primiparous and multiparous cows have different total-tract digestibility of organic matter, crude protein, aNDFom, and slow pool of aNDFom.
• Fair-off and close-up cow diets, which are poor in starch and with a high-forage content, had a lower total-tract starch digestion.
• Primiparous cows yielded 70% of milk that did multiparous cows in the first 45 days of lactation.

## Introduction

The transition period (TP) is one of the most challenging periods for dairy cows. At this time, the cow experiences a series of nutritional (Cavallini et al., 2018), physiological (Buonaiuto et al., 2022), and social changes (Chiesa, Gaiani, Formigoni & Accorsi, 1991; Fustini et al., 2017) and is more vulnerable to infectious and metabolic diseases (Goff & Horst, 1997). Therefore, the proper feeding of these animals to meet their needs and to avoid many important disorders is essential (Cavallini et al., 2021), ensuring the rapid return to positive energy balance and a good reproductive performance (Cabrera, 2014; Formigoni & Trevisi, 2003; Formigoni, Fusaro & Giammarco, 2003; Huzzey, von Keyserlingk & Weary, 2005). One of the most important aspects regarding feeding management is the energy supply, where digestibility of feeds plays a very important role (Formigoni et al., 2003; NASEM, 2021).

Carbohydrates are the primary source of energy in diets fed to dairy cow and usually comprise 60 to 70 percent of the diet. Soluble fiber and water-soluble carbohydrates have the potential to be nearly completely degraded in the rumen, whereas ruminal digestibility of aNDFom and starch are lower and highly variable by source and processing (Ferraretto, Crump & Shaver, 2013; Fustini et al., 2017). Ruminal digestibility of starch and aNDFom are typically lower than soluble fiber and WSC. Moreover starch and aNDFom are highly variable in content (NASEM, 2021). Researchers have noted that both the amount of fiber and its chemical and physical characteristics strongly affect dry matter intake (DMI), chewing activity, cow performance, and milk quality, especially fiber from forage (Bonfante et al., 2016; Fustini et al., 2011, 2017; Grant, 1997; Miller et al., 2021; Oba & Kammes-Main, 2022) Lignification of NDF varies among forages and non-forage fiber sources and is negatively related to digestibility (Van Soest, 1994).

Ruminal starch digestibility is affected by its concentration in the diet (Oba & Allen, 2003a; Voelker & Allen, 2003) as well as intrinsic grain characteristics, mechanical processing and time of ensiling. In vitro starch digestibility (IVSD) of high-moisture corn samples increased by 9 percentage units from October to August of the following year (Ferraretto, Taysom, Taysom, Shaver & Hoffman, 2014). Other factor that affects starch digestibility is processing; greater ruminal and total-tract starch digestibility is well established in dairy cows fed high-moisture corn compared with dry corn (Ferraretto et al., 2013; Firkins, Eastridge, St-Pierre & Noftsger, 2001). Because corn silage and high-moisture corn are harvested before physiological maturity, their degree of vitreousness is less than that of dry shelled corn (Ngonyamo-Majee, Shaver, Coors, Sapienza & Lauer, 2009).

Total-tract digestibility of nutrients is also influenced by animal (e.g. size, DMI) factors, like rumination, and rate of passage (Hanigan, Appuhamy & Gregorini, 2013; Van Amburgh et al., 2015). It is known that there are some differences between primiparous (PP) and multiparous (MP) regarding DMI. Body weight and milk yield account for the biggest differences of this parameter, but there is also the parity component that brings a correction and is considered in the most recent equations (de Souza, Tempelman, Allen & VandeHaar, 2019), although the reason for the parity component still remains unclear. Therefore, we hypothesized that there might be differences also in nutrients’ digestibility among PP and MP cows. The objective of this study was to identify differences on nutrients total-tract digestibility of PP and MP cows during the TP, since a better understanding of these differences would allow dairy farmers and nutritionists to optimize diets to meet the requirements of PP and MP in the TP.

## Materials and methods

### Cows, housing, and diets

A longitudinal observational study was conducted in a commercial dairy ∼15 km northeast of Teramo, Abruzzo, Southern Italy. The protocol was approved by Ethic Committee of the Veterinary Medicine Department of the University of Teramo with the number 18,528/2022.

For this study, 11 PP and 14 MP healthy Holstein cows were selected by age, parity, and body weight. The PP group was represented by animals of age 2.18 ± 0.40 years, body weight (30 days before partum) of 682 ± 30 kg, while MP aged 4.71 ± 1.73 years, body weight (7 days before partum) of 749 ± 33 kg. MP cows had a lactation number of 3.31 ± 1.38, DIM at drying of 337.46  ±  49.04 days, and an average milk yield (MY) on the first 45 days of 46.7  ±  12.5 kg, while the average MY of PP was 32.8  ±  10.3 kg. The groups fair-off (from 60 to 21 days before the expected day of parturition), close-up (from 21 days to calving day) fresh (from calving day until 14 days post-partum) and peak (after 14 days post-partum) (from calving to 30 days after it) were kept divided in different boxes with their respective diets (Table 1), in an open lot facility with 18 m^2^ available per animal; the peak group was housed in a free stall barn, were PP and MP were divided in different pens with 9 m^2^ available per animal; the bedding in all pens consisted in recycled manure solids. Tables 1 and 2 illustrate diet composition and chemical analysis, respectively. The diets were prepared every day using a mixer wagon Matrix Rover Jumbo (Italmix SRL, Italy) and fed ad libitum (daily orts between 3 and 5%) once a day at 0800 h. Cows were milked three times a day, at 0400, 1200, and 1900 h, in a 16-unit herringbone milking parlor. Individual milk production was recorded at each milking in the first 45 days of lactation.

**Table 1:** Composition of the experimental rations fed during the nutritional trial (in kg DM and% of dietary DM).

Ingredients	Fair-off^1^		Close-up^1^		Fresh^1^		Peak^1^	
	kg	%	kg	%	kg	%	kg	%
Corn Silage	–	–	–	–	2.5	12.5	5.4	17.6
Grass Hay	7.8	83.5	7.8	70.8	2.1	10.5	1.5	4.9
Triticale Silage	–	–	–	–	1.6	8.2	1.8	5.9
Alfalfa Hay 17% CP	–	–	–	–	3.2	16.0	3.6	11.8
Wheat Straw	0.45	4.8	0.45	4.1	–	–	0.5	1.6
Cottonseed	–	–	–	–	–	–	1.8	5.9
Soybeans, Ext. 48%	0.9	9.6	0.9	8.2	2.2	11.3	3.5	11.4
Corn Grain, Dry Ground^2^	–	–	1.2	10.8	3.3	16.8	4.8	15.7
Wheat Bran	–	–	–	–	2.0	10.1	2.6	8.6
Corn Grain, Steam-Flaked	–	–	–	–	1.7	8.6	2.6	8.6
Mineral	0.2	2.1	–	–	0.4	1.9	0.5	1.6
Sugar Plus Milker Liq.^3^	–	–	–	–	0.5	2.7	0.9	2.9
Calcium Soap	–	–	–	–	–	–	0.24	0.8
Sodium Bicarbonate	–	–	–	–	0.2	1.0	0.25	0.8
Bio-Chlor^4^	–	–	0.26	2.4	–	–	–	–
Ca Carbonate	–	–	0.06	0.5	–	–	–	–
Corn Gluten Feed	–	–	0.35	3.2	–	–	0.5	1.6
Water	–	–	–	–	0.05	0.4	0.1	0.3
Total	9.4	100.0	11.0	100.0	19.7	100.0	30.6	100.0

1 Dry matter of diets fair-off, close-up, fresh and peak was 92.3, 91.4, 61.6 and 58.4%, respectively.

2 Below the aflatoxin EU maxim tolerable level (Girolami et al., 2022).

3 Composition: Sugarcane molasses, beet pulp molasses, glucose syrup, malted barley, saccarose, sodium chloride.

4 Composition: Condensed corn fermentation solubles, processed grain by-products, condensed extracted glutamic acid fermentation product, magnesium chloride hexahydrate.

**Table 2:** Results of the analysis of major dietary nutrients (% DM) of each experimental ration.

Diet Analisys (% DM)	Fair-off	Close-up	Fresh	Peak
DM	56.17 ± 1.97	56.91 ± 1.96	59.63 ± 1.88	57.27 ± 1.61
CP	13.53 ± 1.37	14.17 ± 1.26	15.78 ± 1.30	16.21 ± 1.10
Ash	8.52 ± 0.71	8.68 ± 0.77	7.31 ± 0.47	7.49 ± 0.30
Starch	9.86 ± 1.32	10.15 ± 0.63	21.66 ± 2.06	22.73 ± 1.17
aNDFom	57.40 ± 4.83	56.28 ± 3.78	37.50 ± 3.05	37.62 ± 2.59
ADF	40.24 ± 6.18	39.05 ± 4.46	27.26 ± 2.61	26.43 ± 1.43
ADL	5.72 ± 0.82	5.72 ± 0.69	4.52 ± 0.23	4.55 ± 0.20
uNDF24	32.68 ± 4.41	31.91 ± 5.06	18.83 ± 2.02	18.71 ± 1.78
uNDF30	32.44 ± 4.05	30.92 ± 4.16	18.01 ± 3.10	17.18 ± 2.52
uNDF120	24.53 ± 1.61	22.28 ± 1.90	16.81 ± 1.91	16.38 ± 1.77
uNDF240	21.15 ± 2.85	20.60 ± 1.62	12.25 ± 1.73	11.95 ± 1.16

DM: dry matter.

CP: crude protein.

OM: organic matter (OM = 100-ash).

*aNDFom: Neutral Detergent Fiber corrected for starch and ash*.

*ADF: Acid Detergent Fiber*.

*ADL: Acid Detergent Lignin*.

*uNDF24: undigestible NDF after 24 h of fermentation*.

*uNDF30: undigestible NDF after 30 h of fermentation*.

*uNDF120: undigestible NDF after 120 h of fermentation*.

*uNDF240: undigestible NDF after 240 h of fermentation*.

### Fecal and feed samples

**Fig. 1:**
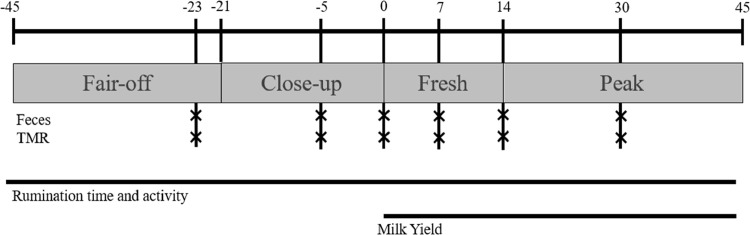
Experimental design implemented in the trial: where the first line (above) is the whole timeline, the boxes indicate the four experimental dietary groups, with respective days of group changing, then, feces and TMR timepoints of collection, finally, rumination time and activity, and milk yield record period.

Fig. 1 is a timeline of the experimental design, with respective sampling and data collection. Fecal and TMR samples were collected 23 ± 3 days before calving, 5  ±  3 days before calving, on day of calving, as well as 7, 14, and 30 after calving. The variation of  ± 3 days for collection on times −23 and −5 happened because of differences between expected and real day of calving. The chosen time points were selected to encompass the complete transition period of dairy cows, spanning from the close-up dry period to the initial month of lactation.

Rectal fecal samples were collected at 0730 h and dried in a stove at 65° for 72 h, milled with a Cyclotec 1093 Sample mill with a 1 mm. The TMR samples were collected over the feed bunk, in the beginning, middle and end, then dried in an oven at 65° for 24 h, and first milled with Retsch MS 100 mill with a 4 mm sieve and then with Cyclotec 1093 mill at 1 mm (Giorgino et al., 2023). After that, about 2 g of dried samples were analyzed by near infrared spectroscopy (NIRS) with Bruker–Tango instruments. Feed and fecal samples were analyzed with a length of wave between 400 and 2498 nm, for the following parameters: crude protein, ash, starch, aNDFom, ADF, ADL, uNDF24 and uNDF240; additionally, uNDF30 and uNDF120 were also analyzed in the TMR. Near infrared spectra (log 1/reflectance) were recorded for each 2 nm range. Applied NIRS calibrations are already published and validated in precedent full paper works (Brogna et al., 2018; Buonaiuto et al., 2021). Nutrients total-tract digestibility (nTTD,%) was calculated according to Ferraretto, Crump, and Shaver (2015) following the same procedure as Cavallini et al. (2023).

### Activity and rumination time

Activity and rumination data were obtained through an ear-tag-based accelerometer (Smartbow, Smartbow GmbH, Weibern, Austria), through 90 days: 45 days before and 45 days after calving. The Smartbow ear-tag consists of an integrated accelerometer that captures data once per second (1 Hz) and sends it in real time to a local server. The milking parlor was equipped with Metatron electronic milk metres, P21 bail controllers, and electronic identification tags (Metatron DairyPlan, GEA GmbH).

### Statistical analysis

All data were analyzed using the JMP pro v 16 statistical program (SAS, NY). The normal distribution of the data was verified using the Shapiro-Wilk test. Mixed models were used for data analysis. The continuously recorded parameters (rumination, activity, and production) were analyzed with a model whose fixed effects are time (from −45 to +45 days from calving), parity (PP or MP) and their interaction (time * parity). The parameters recorded at specific time points (feces analysis and digestibility indexes) were analyzed with a model whose fixed effects are the sampling time point (−23, −5, 0, 7, 14, 30 days from calving), the parity (PP or MP) and their interaction (time * parity). The single cow, associated with the day of lactation and parity, was considered the experimental unit and included as a random effect. Obtained model residuals were then checked for normality. In the tables and graphs, the results are expressed as the least squares mean and the standard error of the mean.

The results were considered tendencies for *p* ≤ 0.10. Significant for *p* ≤ 0.05 and very significant for *p* ≤ 0.01. Tukey's test was used to analyze the differences in the single timepoints if the interaction between the fixed effects was tendential or significant.

## Results

**Fig. 2:**
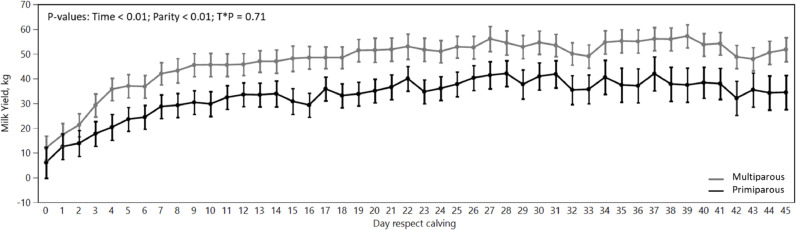
Evolution of milk yield ( kg) of primiparous (PP) and multiparous (MP) cows on the first 45 days of lactation.

The objective of this study was to evaluate the differences in nutrients total-tract digestibility of PP and MP cows during the TP. The composition of the experimental rations fed during the nutritional trial are shown in Table 1. Results of the analysis of major dietary nutrients of each experimental ration are shown in Table 2. Milk yield (Fig. 2) in PP cows was about 70% than yield of MP cows (32.8  ±  10.3 vs. 46.7  ±  12.5).

**Fig. 3:**
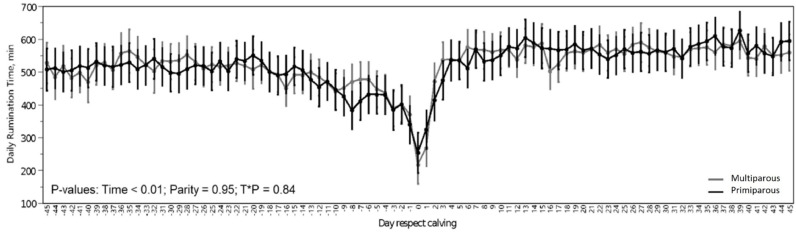
Evolution of daily rumination time ( min) for primiparous (PP) and multiparous (MP) from the 45 days before and after calving.

Rumination time (Fig. 3) was at a normal range for healthy dairy cattle with a total average of 514 min in 24 h. There was significant decrease on rumination time (*p <* 0.01; 360 min in 24 h) in the day of calving, while no differences in daily rumination time (*p* = 0.92) were found between PP and MP.

The results reported about feces composition (Table 3) showed a significant difference between PP and MP for DM (*p <* 0.01), CP (*p* = 0.01), ADF (*p* = 0.05), uNDF_240 (_*p* = 0.01), pdNDF_240_ (*p* = 0.05), and a tendency for aNDFom (*p* = 0.09), which then reflected differences also in the total-tract digestibility of that nutrients.

**Table 3:** Results of the feces analysis (% DM) for primiparous (PP) and multiparous (MP) cows in the sampled time points.

	T-23		T-5		T0		T7		T14		T30		*p-value*			
	MP	PP	MP	PP	MP	PP	MP	PP	MP	PP	MP	PP	SEM	Time	Parity	T*P
DM	13.69	15.23	13.57	14.81	13.75	15.76	14.52	15.90	14.84	15.66	16.25	16.84	0.44	< 0.01	< 0.01	0.36
OM	87.58	87.98	87.57	87.98	87.61	88.09	87.96	88.38	87.92	88.23	87.91	88.47	0.38	0.51	0.19	0.99
CP	11.83	13.21	12.09	13.19	12.18	13.03	11.78	13.50	12.11	13.83	12.64	13.32	0.54	0.79	0.01	0.70
Ash	12.42	12.02	12.43	12.02	12.39	11.91	12.04	11.62	12.08	11.77	12.09	11.53	0.38	0.51	0.19	0.99
Starch	1.63	1.82	1.78	1.96	1.76	2.05	1.63	2.18	1.52	2.16	1.63	2.24	0.27	0.92	0.11	0.74
aNDFom	62.15	59.88	60.68	60.15	60.84	60.18	62.13	59.34	61.80	58.82	61.28	59.62	1.16	0.97	0.09	0.61
ADF	47.16	44.79	46.72	44.32	46.71	44.74	46.92	43.88	46.95	43.66	46.22	43.83	1.28	0.93	0.05	0.98
ADL	21.26	19.12	21.07	19.35	20.65	19.99	20.74	19.01	21.07	19.09	20.75	18.69	1.18	0.98	0.16	0.95
uNDF24	54.75	53.31	53.54	52.89	53.87	53.27	54.76	52.56	54.76	51.99	54.11	52.72	0.95	0.91	0.10	0.65
uNDF240	45.18	42.67	45.15	41.64	45.32	42.23	45.20	41.99	44.67	41.60	44.67	41.87	1.13	0.91	0.01	0.99
pdNDF24	7.40	6.57	7.33	6.81	6.89	6.91	7.37	6.78	7.13	6.89	7.18	6.90	0.34	0.99	0.10	0.75
pdNDF240	16.97	17.20	15.49	18.07	15.30	17.95	16.93	17.36	17.13	17.25	16.61	17.74	0.80	0.94	0.05	0.21

DM: dry matter.

OM: organic matter (OM = 100-ash).

CP: crude protein.

*aNDFom: Neutral Detergent Fiber corrected for starch and ash*.

*ADF: Acid Detergent Fiber*.

*ADL: Acid Detergent Lignin*.

*uNDF24: undigestible NDF after 24 h of fermentation*.

*pdNDF24: potentially digestible NDF after 24 h of fermentation*.

*uNDF240: undigestible NDF after 240 h of fermentation*.

*pdNDF240: potentially digestible NDF after 240 h of fermentation*.

**Fig. 4:**
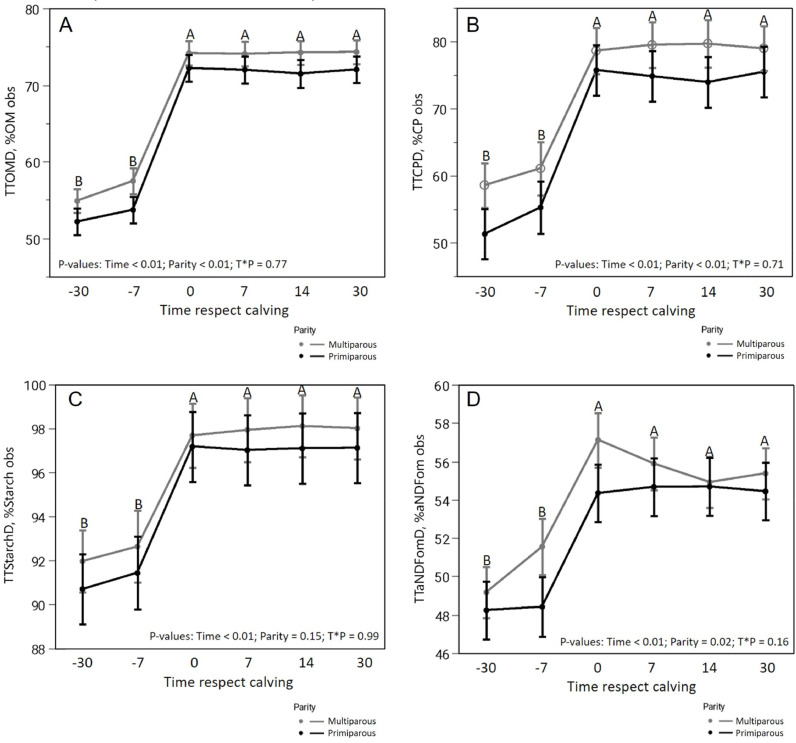
Evolution of the observed total-tract digestibility (%) among sampled timepoints and parity (primiparous, PP vs multiparous, MP) for organic matter (TTOMD, chart A), crude protein (TTCPD, chart B), starch (TTstarchD, chart C), and aNDFom (TTaNDFomD, chart D).

Data regarding total-tract digestibility (Table 4, Fig. 4) for PP and MP show that nTTD were different (*p* ≤ 0.02) for aNDFom and pdNDF_240_ (52.5 vs. 54.0 and 78.8 vs. 81.3, respectively), while no differences were found regarding pdNDF_24_ and starch (88.5 vs. 88.6 and 95.1 vs. 96.1, respectively). There were differences among timepoints for all nutrients analysed for TTD (*p <* 0.01, Table 4).

**Table 4:** Results of the total-tract digestibility (TTD,%DM) in the analyzed nutrients of primiparous (PP) and multiparous (MP) cows in the sampled time points.

	T-23		T-5		T0		T7		T14		T30		*p-value*			
TTD	MP	PR	MP	PR	MP	PR	MP	PR	MP	PR	MP	PR	SEM	Time	Parity	T*P
**OM**	54.89	52.18	57.49	53.71	74.17	72.22	74.07	72.00	74.24	71.48	74.32	72.03	0.91	< 0.01	< 0.01	0.77
**CP**	58.55	51.30	61.03	55.24	78.59	75.71	79.48	74.81	79.67	73.93	78.96	75.47	2.00	< 0.01	< 0.01	0.71
**Starch**	91.96	90.70	92.63	91.43	97.68	97.18	97.94	97.02	98.11	97.09	98.00	97.11	0.84	< 0.01	0.15	0.99
**NDF**	49.17	48.24	51.56	48.42	57.12	54.35	55.89	54.67	54.92	54.69	55.37	54.44	0.79	< 0.01	0.02	0.16
**ADF**	45.14	44.79	45.79	44.23	53.80	52.39	53.24	52.92	52.57	52.41	53.35	52.53	0.89	< 0.01	0.27	0.92
**pd24**	85.92	86.83	86.09	86.10	90.46	89.69	89.80	89.75	89.70	89.24	89.57	89.23	0.55	< 0.01	0.73	0.61
**pd240**	77.87	76.38	80.50	75.60	83.97	79.90	82.15	80.35	81.32	80.31	81.99	80.00	1.19	< 0.01	0.01	0.27

TT: Total-tract digestibility.

OM: TTOMD,% OM.

SD: TTstarchD,% Starch.

CP: TTCPD,% CP.

NDF: TTaNDFomD,% aNDFom.

ADF: TTADFD,% ADF.

pd24: TTpdNDF_24_D,% pdNDF_24_.

pd240: TTpdNDF_240_D,% pdNDF_240_.

SEM: Standard Error of Mean.

T*P: Time*Parity.

## Discussion

Data regarding diets composition, feces analysis and total-tract digestibility are reported on Tables 1 to 4. The diets of the entire experimental periods cover the dairy cow requirements (Tables 1 and 2) in agreement to NASEM (2021) recommendations, and are typical in most intensive dairy production systems in Italy (Serva, Magrin, Andrighetto & Marchesini, 2021; Zucali et al., 2018), where forages used in the TMR are mostly corn silage, small grains silage, and hay.

The milk yield (Fig. 2) difference of PP compared to MP cows was about 30%, which is slightly lower than data from the literature (Azizi, Kaufmann & Hasselmann, 2009; Wathes et al., 2007). Since grouping is a component of the cow's feeding environment that can modulate intake as a result of its impact on cow comfort, competition for feed and other resources, and herd health (Bach, Iglesias, Devant & Ràfols, 2006; Grant & Albright, 2001), keeping the animals in the same pen during the first 14 days post-partum could negatively affect PP cows performance.

Rumination time (Fig. 3) was at a normal range for healthy animals (Soriani, Trevisi & Calamari, 2012). The significant decrease on rumination time in the day of calving was expected as it has already been evidenced in other works (Macmillan, Gobikrushanth & Colazo, 2022; Pahl, Hartung, Grothmann, Mahlkow-Nerge & Haeussermann, 2014). No differences in daily rumination time were found between PP and MP (Fig. 3).

Data regarding total-tract digestibility (Table 4, Fig. 4) for PP and MP show that nTTD were different for aNDFom and pdNDF_240_ (52.5 vs. 54.0 and 78.8 vs. 81.3, respectively), while no differences were found regarding pdNDF_24_ and starch (88.5 vs. 88.6 and 95.1 vs. 96.1, respectively). As shown in previous studies, cow size and parity affect total-tract digestibility and DMI (de Souza et al., 2019; NASEM, 2021). A higher dry matter intake decreased mainly TTpdNDF_240_ on heifers fed diets with a same composition, but different in physical form (pellet vs. TMR; Bonfante et al., 2016). These results evidenced how DMI influences passage rate, which can negatively affect the fraction of NDF that is potentially digestible but needs a longer time, in other words, the slow degradable portion of pdNDF. TTpdNDF_240_D on this study could be higher than other studies likely because of the feedstuffs used in the ration: e.g. the main forage used in our study was corn silage and grass hay, which have a higher digestible fraction and lower passage rate than alfalfa-hay based diets (Fustini et al., 2017; Miller et al., 2021). Furthermore, fecal sampling results can also be affected by the time of it is collected, regarding the hours after feeding (Cavallini et al., 2023), but it was not a problem in this study since all sampling were done always in the early morning.

The TTaNDFom could be explained by the higher inclusion and poorer quality of forage for groups fair-off and close-up when compared to fresh and high producing cows, since different inclusions of feedstuffs can impact the total-tract digestibility (Cavallini et al., 2023). The TTaNDFom depends on its intrinsic characteristics, that affect the maximal rate and extent of digestion, retention time in the fermentation compartments, and concentrations and activity of microbial enzymes (Allen & Mertens, 1988). The pattern of results for TTaNDFomD on this study is like the one found by Miller et al. (2021), where just lactating cows were used, and total-tract digestibility of aNDFom was of 55.7% for a low NDF ration, while 48.9% for a high NDF ration (DM basis).

The TTstarchD differences among timepoints (*p <* 0.01), which went from 91.33 to 97.55% of dietary total starch from T-23 to T30, can be explained by the different starch concentration of diets, which leads to different ruminal environments regarding the capacity of digesting starch. When comparing groups fair-off and close-up with fresh and peak groups, dietary starch level is very different. As showed by Oba and Allen (2003b) in lactating cows, the fractional rate of starch digestion as well as ruminal digestibility of starch increased when corn grain was substituted for fNDF or non-forage fiber sources (beet pulp; Voelker & Allen, 2003). Ruminal contents of cows fed high- vs. low-starch (32 vs. 21% DM) diets may have insufficient amylolytic activity for maximal starch digestion when readily fermentable starch is available (Oba & Allen, 2003a), leading to such a point that a post ruminal digestibility does not totally compensate it (Callison, Firkins, Eastridge & Hull, 2001), as it can be seen among non-lactating and lactating cows of our study. Greater starch digestibility with higher-starch diets indicates that starch degradability in the rumen is a function of both the source as well as characteristics of the microbial population in the rumen (NASEM, 2021).

Differences in TTCPD among timepoints were probably due to the source of protein, since protein from fair off and close-up groups come greater from grass hay, which is less degradable than protein grain sources (Chrenková et al., 2014; Ghoorchi & Arbabi, 2010).There was also difference regarding DM content of feces among timepoints and among PR and MP groups (Table 3). DM differences of feces among timepoints could be explained by different passage rates (NASEM, 2021) and water intake through the lactation cycle, since diets have many differences factors that could modulate water intake, such as dietary protein, NDF, Na and K content (Appuhamy, Judy, Kebreab & Kononoff, 2016). There is insufficient data in literature to explain differences in feces DM of PR and MP; it could be the development status of the gastrointestinal tract of PP, which could be less efficient in water reabsorption, but studies should be done to support that.

The TTD of sugars were not analysed since it is well known that this fraction of carbohydrates has a very high degradation rate and has the potential to be nearly completely degraded in the rumen. In fact, degradation of sucrose, fructose and glucose (the major sugars in feedstuffs) had a ruminal degradation rate that goes from 50% h-1 to 250% h-1 (Weisbjerg, Hvelplund & Bibby, 1998).

We did not observe differences between PR and MP regarding TTpdNDF_24_D (*P* = 0.73), while a significant difference for TTpdNDF_240_D (*P* = 0.01) is reported. Possible reasons for these results among younger and older cows might be due to differences in rumen volume, as it has been well established in herbivores that there is a strong relationship between BW and gastrointestinal capacity (Azizi, Hasselmann & Kaufmann, 2010; Van Soest, 1994). Since PR cows have a smaller rumen volume, their passage rate is probably faster, which affects the total digestion of the slow portion of pdNDF (pdNDF_240_), but not the fast portion pdNDF_24_. Moreover, it can be assumed that this difference is basically due to rumen volume, since is stated in literature that ruminal digestibility of NDF accounts for over 90 percent of TTaNDFom (Huhtanen, Ahvenjärvi, Broderick, Reynal & Shingfield, 2010).

No differences regarding TTstarchD were related to parity (*P* = 0.15). TTstarchD is not affected by ruminal volume probably because of intestinal digestion and its intrinsic faster k_d_ compared to fibrous fractions (NASEM, 2021). As showed by Ferraretto et al. (2013), even when the source of starch changes from a faster to a slower digestion rate, the TTstarchD remains unchanged; based on a meta-analysis that considered ruminal and TTstarchD, ruminal digestibility of starch was greater for wheat (79% of intake) and barley (71% of intake) than for corn (54% of intake), whereas TTstarchD did not differ (93 to 94% of intake).

TTCPD changes among PR and MP. It could be related to a higher passage rate, which leads to a higher B and C fraction of protein that escapes from digestion and passes through the gastrointestinal tract. B fraction is potentially degradable in the reticulorumen (Lanzas, Tedeschi, Seo & Fox, 2007), but a higher passage rate could negatively interfere with protein digestion due to the smaller volume of reticulorumen of PR cows. Another explanation for this might be that the gastrointestinal tract of PR cows could be in a development phase, so it still would not have achieved the maximum potential of digestion, or it could be shorter than MP cows but, to our knowledges, there is no experimental data to support that. Besides gastrointestinal tissue development, the rumen microbiome might play an important role in explaining these results since the rumen microbiome can be in development and adaptation as animals reach the adulthood (Jami, Israel, Kotser & Mizrahi, 2013) and is dynamic in terms of diversity on the course of lactations (Jewell, McCormick, Odt, Weimer & Suen, 2015). Furthermore, the rumen microbiome changes a lot as diet changes (Zhu et al., 2018), hence it could be more challenging to PP because heifers’ diets are much more different than a diet for late lactation cows.

Some limitations of this study include that it was not possible having DMI data because animals were in a group. Anyway, based on feed delivered and orts we could get the group´s DMI. Feces collections were made in just one time of the day, but we kept the standardization of collecting it always at the same time of the day.

As practical applications of this study, we can cite that this is showing important differences among PP and MP regarding total-tract digestion of nutrients. Since PP do not have the same ability of digesting nutrients, a little raise in ration density of nutrients such as CP and starch fractions might be considered to compensate it, and better meet their requirements.

## Conclusions

TTstarchD was different among timepoints, going from an average of 91.40 up to 97.39% of starch, on times −23 and 14, respectively. Differences in TTD of NDF among timepoints was expected because of differences in diet composition among lactating and non-lactating cows.

The slow portion of aNDFom (pdNDF_240_) and total aNDFom was significantly less digestible in PP compared to MP, with averages of TTpdNDF_240_D ranging from 75.80 to 80.50% of aNDFom for PP, and from 77.87 to 83.97% of aNDFom for MP.

With a similar behavior as presented for the slow portion and total aNDFomD, PP and MP cows also had different TTOMD, which averages ranged from 52.18 to 72.22% of OM for PP, and from 54.89 to 74.32% of OM to MP.

These differences could have an important impact on energy and protein supply for PP, which show a lower digesting capacity. They should be taken into consideration when formulating diets.

## Availability of data

The data that support the findings of this study are available from the corresponding author, DC, upon reasonable request.

## Ethical statement

The protocol was approved by Ethic Committee of the Veterinary Medicine Department of the University of Teramo with the number 18,528/2022.

## Declaration of Competing Interest

The authors declare that they have no known competing financial interests or personal relationships that could have appeared to influence the work reported in this paper.
